# Comparative study of transepidermal water loss in patients with and without hyperhidrosis by closed-chamber measurer in an air-conditioned environment

**DOI:** 10.31744/einstein_journal/2018AO4312

**Published:** 2018-10-30

**Authors:** Andre Miotto, Pedro Augusto Antunes Honda, Thiago Gangi Bachichi, Caio Santos Holanda, Ernesto Evangelista, João Alessio Juliano Perfeito, Luiz Eduardo Villaça Leão, Altair da Silva Costa

**Affiliations:** 1 Escola Paulista de Medicina, Universidade Federal de São Paulo, São Paulo, SP, Brazil.; 2 Hospital Israelita Albert Einstein, São Paulo, SP, Brazil.

**Keywords:** Thoracic surgery, Hyperhidrosis, Sympathectomy, Cirurgia torácica, Hiperidrose, Simpatectomia

## Abstract

**Objective:**

To evaluate the difference in transepidermal water loss in patients diagnosed with hyperhidrosis and healthy subjects, in an air-conditioned environment.

**Methods:**

Twenty patients diagnosed with hyperhidrosis and 20 healthy subjects were subjected to quantitative assessment using a closed-chamber device, in six previously established sites.

**Results:**

The measurements showed different transepidermal water loss values for healthy subjects and patients with hyperhidrosis, especially in the hands and feet. In the Control Group, the median for the hands was 46.4g/m^2^/hour (p25: 36.0; p75: 57.6), while in the Hyperhidrosis Group, the median was 123.5g/m^2^/hour (p25: 54.3; p75: 161.2) – p<0.001. For the feet, the Control Group had a median of 41.5g/m^2^/hour (p25: 31.3; p75: 63.5) and the Hyperhidrosis Group, 61.2g/m^2^/hour (p25: 32.3; p75: 117) – p<0.02. Measurements of the axillas also showed differences. In the Control Group, the median was 14.8g/m^2^/hour (p25: 11.8; p75: 19.0) and, in the Hyperhidrosis Group, 83.5g/m^2^/hour (p25: 29.5; p75: 161.7) – p<0.001.

**Conclusion:**

Measuring transepidermal water loss is sufficient for diagnosis and follow-up of patients with hyperhidrosis.

## RESUMO

**Objetivo:**

Avaliar a diferença entre a perda transepidérmica de água aferida entre pacientes com e sem diagnóstico de hiperidrose, em ambiente climatizado.

**Métodos:**

Foram selecionados 20 pacientes com diagnóstico de hiperidrose e 20 hígidos, submetidos à aferição de maneira quantitativa, com mensurador de câmara fechada, em seis locais previamente estabelecidos.

**Resultados:**

As medidas realizadas mostraram valores diferentes de perda transepidérmica de água em pessoas hígidas e naquelas com hiperidrose, principalmente em mãos e pés. No Grupo Controle, a mediana das aferições em mãos foi 46,4g/m^2^/hora (p25: 36,0; p75: 57,6), enquanto, no Grupo Hiperidrose, obtivemos a mediana de 123,5g/m^2^/hora (p25: 54,3; p75: 161,2) – p<0,001. Já nos pés, a mediana no Grupo Controle foi 41,5g/m^2^/hora (p25: 31,3; p75: 63,5) e, no Grupo Hiperidrose, foi 61,2g/m^2^/hora (p25: 32,3; p75: 117) – p<0,02. As medidas das regiões axilares também mostraram diferença. No Grupo Controle, obtivemos mediana 14,8g/m^2^/hora (p25: 11,8; p75: 19,0) e, no Hiperidrose, 83,5g/m^2^/hora (p25: 29,5; p75: 161,7) – p<0,001.

**Conclusão:**

A mensuração da perda transepidérmica de água é suficiente para diagnóstico e acompanhamento de pacientes com hiperidrose.

## INTRODUCTION

Hyperhidrosis is a relatively frequent dysfunction, with an incidence of 0.6 to 1% of population. It can be classified as generalized and focal, and the latter is characterized by excessive sweating, bilaterally and symmetrically, at certain sites of the body, with no relation with the need for heat loss. It is more frequent in young adults and adolescents, and predominant among women.^(^ ^1^ ^-^ ^3^ ^)^ Primary focal hyperhidrosis is the most frequent indication for thoracic sympathectomy, which improves 80 to 90% of cases.

The diagnosis of hyperhidrosis is subjective and varies based on each patient’s complaint. Hyperhidrosis affects social, professional and leisure activities, with a negative impact on quality of life. Tools have been developed to transform the subjective evaluation in a measurable parameter, such as specific quality of life questionnaires. Another tool is the objective measurement of sweating, using the rate of water evaporation through the skin, known as “transepidermal water loss” (TEWL). Quantifying this loss of water can be useful for diagnosis, post-operative follow-up and evaluation of compensatory sweating after sympathectomy.^(^ ^4^ ^,^ ^5^ ^)^

Different tools have been developed to quantitatively measure TEWL, as set forth in protocols published by the European Society of Contact Dermatitis (ESCD) and the European Group on Efficacy Measurements of Cosmetics and other Topical Products (EEMCO). The most widely used today are closed-chamber and open-chamber water flow meters. Closed-chamber meters are known for being easier to use than open-chamber devices.

In TEWL studies, it is important to establish reference values, both for patients diagnosed with hyperhidrosis and subjects in the Control Group. These values can change according to climate, population and location in which the study is conducted. Few studies have described normal values for sweating, and those under controlled temperatures are even scarcer.^(^ ^2^ ^,^ ^5^ ^-^ ^7^ ^)^

The importance of this article is in establishing reference values for future studies, besides proving the effectiveness of objective measurement as a method for diagnosis and post-operative follow-up of hyperhidrosis, even in temperature controlled environments.^(^ ^7^ ^)^

## OBJECTIVE

To evaluate the difference in transepidermal water loss between patients diagnosed with hyperhidrosis and healthy subjects in an air-conditioned environment.

## METHODS

Case-control study of healthy subjects and patients diagnosed with palmar and/or axillary hyperhidrosis, evaluated by the Thoracic Surgery Department of *Hospital São Paulo da Escola Paulista de Medicina da Universidade Federal de São Paulo* , between August 2014 and March 2015. We enrolled 40 subjects, of which 20 were in the Hyperhidrosis Group and 20 in the Control Group.

For the Hyperhidrosis Group, we selected patients with bilateral, symmetric, focal exacerbated sweating for at least 6 months, with some level of limitation of daily activities. For the Control Group, we selected healthy, asymptomatic subjects with no morbidities, no complaint of excessive sweating and no family history of hyperhidrosis.

We excluded individuals with a body mass index ≥25kg/m^2^, other morbidities, pregnant women, aged under 14 or over 40 years, generalized or craniofacial hyperhidrosis, smokers, with active infectious diseases or those who did not agree to take part in the study.

All patients assessed were subjected to TEWL measurement using the VapoMeter^®^ closed-chamber device (Delfin Technologies Ltd, Kuopio, Finland) at six previously established sites:^(^ ^5^ ^,^ ^7^ ^)^ palms, soles, axillae, mid-anterior chest region (at the level of the xyphoid process), lumbar region and infraumbilical abdomen. The device used in this study is portable and practical, and was previously used in other studies with proven results.^(^ ^5^ ^-^ ^10^ ^)^

The skin of subjects and controls was intact, with no lesions at the measurement sites. Measurements were taken in a room with a temperature between 18 and 19°C, and relative humidity between 48 and 50%.^(^ ^11^ ^)^

Patients rested in the room for 10 to 20 minutes before the measurement, for acclimatization. They were instructed not to use any products on the skin on the day of measurement (for at least 12 hours) and not to perform any physical activity before the evaluation.^(^ ^7^ ^,^ ^10^ ^)^ All measurements were taken in the morning by the same examiner, previously trained on how to use the meter.

We calculated the sample size for the study design with the 30% difference expected in the results, and the minimum number was defined as 19 subjects per group. The statistical analysis used the Student’s *t* test for normal distribution variables, and the Shapiro-Wills or the Wilcoxon-Mann-Whitney test for asymmetric distribution variables.

This study was approved by the Institutional Review Board of the *Universidade Federal de São Paulo* , under number 554.150, CAAE: 23156614.1.0000.5505.

## RESULTS

All subjects were evaluated for skin type (Fitzpatrick scale) and fell between I and IV, even though there were no restrictions to types V and VI.

The mean body mass index in the Control Group was 23.02 (±1.42) and in the Hyperhidrosis Group, 21.78 (±2.17). In the Control Group, the measurement was taken in a room at 18.00°C (±0.00), with a relative humidity of 48.00% (±2.42). The Hyperhidrosis Group was measured at 18.25°C (±0.43), with a relative humidity of 49.05% (±4.66). We analyzed the null hypothesis for the groups before measuring the reported variables. There were no differences between the groups in the parameters measured by the Student’s *t* test.

The analysis was conducted in an air-conditioned room and at the most frequent sites affected by hyperhidrosis: hands, feet and axillae. We found significant differences for all sites ( Tables 1 and 2 ).

**Table 1:** Comparison of transepidermal water loss values in hands, axilla and feet, measured using VapoMeter® in an air-conditioned environment

Site	Control Group			Hyperidrosis Group			p value
	
	Median	p25	p75	Median	p25	p75	
Right hand	46.4	36.0	57.6	123.5	54.3	161.2	0.001
Left hand	41.4	31.2	54.0	111.5	42.5	137.7	0.001
Right foot	41.5	31.3	63.5	61.2	38.6	117.0	0.023
Left foot	41.5	31.8	61.2	64.9	41.3	110.0	0.033
Right axilla	14.8	11.8	19.0	83.5	29.5	161.7	0.001
Left axilla	13.7	12.3	16.2	76.9	38.5	162.0	0.001

Mann-Whitney U test. Measures in g/m^2^/hour.

**Table 2:** Comparison of transepidermal water loss values in both hands, axilla and feet, measured using VapoMeter® in an air-conditioned environment

Site	Control Group			Hyperidrosis Group			p value
	
	Median	p25	p75	Median	p25	p75	
Hands	46.4	36.0	57.6	123.5	54.3	161.2	0.001
Feet	41.5	31.3	63.5	61.2	38.6	117.0	0.023
Axilla	14.8	11.8	19.0	83.5	29.5	161.7	0.001

Mann-Whitney U test. Measures in g/m^2^/hour.

When measuring at the sites most frequently affected by compensatory sweating (anterior chest region, lumbar region and abdomen), only one site showed a statistically significant difference ( Table 3 ).

**Table 3:** Comparison of median values for transepidermal water loss in others sites, measured using VapoMeter® in an air-conditioned environment

Site	Control Group			Hyperidrosis Group			p value
	
	Median	p25	p75	Median	p25	p75	
Sternum	12.5	9.7	14.6	13.2	10.6	16.2	0.32
Lumbar	11.9	10.8	14.6	11.5	10.2	15.1	0.94
Abdomen	13.4	11.2	17.4	16.0	13.6	23.7	0.04

Mann-Whitney U test. Measures in g/m^2^/hour.

## DISCUSSION

Most patients with hyperhidrosis are seen by thoracic surgery services and referred for surgery based only on the complaint. Other facilities evaluate them using quality-of-life questionnaires targeted at hyperhidrosis, also used for postoperative comparison.^(^ ^12^ ^,^ ^13^ ^)^ Objective quantification of hyperhidrosis is rarely used, perhaps because it takes time, has no established relation with diagnosis or treatment of the condition, and has high costs. Nevertheless, it is a useful tool for clinical studies and comparison of pre- and postoperative results, in addition to allowing for documentation and recording.^(^ ^2^ ^,^ ^5^ ^,^ ^7^ ^)^

In this study, we used a closed-chamber device - VapoMeter^®^, also used in other studies, due to its easiness of use and accuracy in quantifying hyperhidrosis. Because it is easy to handle and portable, it can be used for pre- and postoperative comparison in the outpatient setting.^(^ ^2^ ^,^ ^3^ ^,^ ^9^ ^)^ Steiner et al., compared the use of open-chamber and closed-chamber devices and concluded that, despite the advantages of each, the results were not comparable due to the different form of measurement.^(^ ^10^ ^)^

Transepidermal water loss can be affected by different factors, such as smoking, obesity, diabetes and others.^(^ ^14^ ^,^ ^15^ ^)^ For this reason, subjects with these conditions were excluded. Exogenous factors that affect TEWL, such as patients’ diets and occupations, were not considered in this study, because they do not change the diagnosis and the sympathectomy indication. Nonetheless, they can affect TEWL results at a smaller scale.^(^ ^14^ ^)^ The skin type can also affect TEWL measurements, and Fitzpatrick V/VI skin types have an epidermal barrier which is more resistant to water loss.^(^ ^16^ ^)^ We did not have any subjects with these skin types in this study. Sex does not affect TEWL.^(^ ^14^ ^,^ ^16^ ^)^

The measurements performed with the VapoMeter^®^ in our study showed different values of TEWL for healthy subjects and patients with hyperhidrosis. Ishy et al.,^(^ ^7^ ^)^ found different values in measurements using the same type of device, before and after sympathectomy. The mean values found in postoperative patients and healthy subjects were comparable. The median value for the hands was 46.4g/m^2^/hour, while Ishy et al.,^(^ ^7^ ^)^ found mean values of 45.75 to 30.81g/m^2^/hour, one month after surgery. In the feet, the median value was 41.5g/m^2^/hour, while they found 66.44 to 53.50g/m^2^/hour. Our measurements in the axillae were unprecedented and indicated differences between the groups.^(^ ^5^ ^,^ ^7^ ^)^ We understand from these results that TEWL measurement using the VapoMeter^®^ can be used as a quantitative and objective tool to diagnose hyperhidrosis in an air-conditioned environment.

The variations of median TEWL values measured in the Control Group compared to the Hyperhidrosis Group were 140% in the hands; 72% in the feet and 490% in the axillae. The chest and lumbar regions did not show any differences between patients with hyperhidrosis and controls. This could be because hyperhidrosis is not evident at these sites, and it more noticeable on the hands, feet and axillae. Chest regions become objects of evaluation and study after thoracic sympathectomy, due to the presence of compensatory sweating in some cases. Before surgery, the differences between the groups are not significant on the clinical examination, because the complaints that lead to the sympathectomy indication are localized sweating on the hands and axillae.^(^ ^5^ ^,^ ^7^ ^,^ ^12^ ^,^ ^13^ ^)^

The results presented also help standardize the room temperature for TEWL measurement. When reviewing the literature, we found several studies using the VapoMeter^®^ as the primary measurement tool, but there is no standardization of the room temperature for the measurements, despite the concern with its control.^(^ ^1^ ^,^ ^4^ ^,^ ^6^ ^,^ ^8^ ^-^ ^10^ ^)^ Only Singh et al., observed a difference in the sweating pattern according to the room temperature. However, before this study, there was no comparison study or definition of a cutoff point that could lead to changes in the values measured.^(^ ^11^ ^)^ We believe that, by standardizing the temperature, we can improve the reliability of TEWL measurements, as well as diagnosis of hyperhidrosis.

Despite the small number of patients and controls assessed, this study was sufficient to prove the hypothesis proposed. Future studies with a larger number of subjects may confirm our results. We emphasize the importance of room temperature and humidity for understanding and specific assessment of hyperhidrosis, particularly in tropical countries like Brazil.

## CONCLUSION

There are differences in the values of transepidermal water loss, particularly in the hands, feet and axillae, between subjects with and without hyperhidrosis, in an air-conditioned environment.
